# Utility of MRI in low and low to moderate density breasts with invasive lobular carcinoma

**DOI:** 10.1186/bcr3250

**Published:** 2012-11-09

**Authors:** B Rengabashyam, N Sharma, B Dall

**Affiliations:** 1 The Leeds Teaching Hospitals NHS Trust, Leeds, UK

## Objective

To determine the feasibility of excluding MRI from the preoperative diagnostic pathway of invasive lobular carcinoma (ILC) in women with low and low to moderate density breasts on mammography.

## Methods

A total of 179 cases of ILC were diagnosed between 2009 and 2012. Forty-eight cases were identified as low and low to moderate density breasts. The study group includes 32 cases who underwent MRI. Parameters scrutinised include size and number of lesions on mammography, ultrasound and MRI, second-look ultrasound, type of surgery, further surgery and histology.

## Results

Twenty-nine cases had low to moderate density breasts and three had purely low density breasts. Average age of women was 64. Size of lesions ranged between 2 and 50 mm with an average of 20.14 mm. In 25/32 cases (78.12%) conventional imaging matched MRI. MRI identified additional disease in 7/32 (21.8%). This was predominantly in the form of satellites around the index lesion resulting in multifocality in 6/7. Four resulted appropriately in mastectomy. Two led to wider WLE appropriately. In one case, multicentric disease was correctly detected and subjected to mastectomy. Second-look ultrasound was recommended in 4/7 cases. All these cases had low to moderate density breasts on mammography and 6/7 cases measured more than 15 mm in size. Ultrasound matched MRI in one mammographically occult case and was subjected to appropriate WLE. In two cases there was much more disease than anticipated from conventional imaging and MRI (6.25%).

## Conclusion

Even in low and low to moderate density breasts where mammography has a higher exclusion value, MRI identified additional disease in 21.8% (7/32).

